# Botulinum Toxin Treatment for Uncommon Phenotypes of Laryngeal Adductor Breathing Dystonia

**DOI:** 10.3390/toxins18060272

**Published:** 2026-06-20

**Authors:** Domenico Antonio Restivo, Angelo Alito, Demetrio Milardi, Mario Stampanoni Bassi, Sara Lanza, Angelo Quartarone, Rosario Marchese-Ragona

**Affiliations:** 1 Department of Clinical and Experimental Medicine, University of Messina, 98125 Messina, Italy; drestivo@unime.it; 2 Brain Mapping Lab, Department of Biomedical, Dental Sciences and Morphological and Functional Imaging, University of Messina, 98125 Messina, Italy; alitoa@unime.it (A.A.); dmilardi@unime.it (D.M.); 3 Department of Biomedical, Dental Sciences and Morphological and Functional Imaging, University of Messina, 98125 Messina, Italy; 4 Unit of Neurology, IRCCS Neuromed, 86077 Pozzilli, Italy; m.stampanonibassi@gmail.com; 5 Faculty of Psychology, Uninettuno Telematic international University, 00186 Rome, Italy; 6 Physical and Rehabilitation Medicine Unit, ASP Ragusa, 97100 Ragusa, Italy; sara.lanza@asp.rg.it; 7 IRCCS Centro Neurolesi Bonino-Pulejo, 98124 Messina, Italy; 8 Department of Neurosciences, Otolaryngology Section, University of Padova, 35122 Padova, Italy; rosario.marcheseragona@unipd.it

**Keywords:** botulinum toxin (BoNT/A), differential diagnosis, Inducible laryngeal obstruction (ILO), inspiratory stridor, laryngeal adductor breathing dystonia (LABD), paradoxical vocal fold adduction, respiratory dystonia

## Abstract

Laryngeal adductor breathing dystonia (LABD) is a rare form of focal, task-specific respiratory dystonia affecting the laryngeal muscles of unknown aetiology. Unlike classical laryngeal dystonia (spasmodic dysphonia), LABD is not primarily characterised by impaired speech, but rather by dysfunction of respiratory laryngeal control. The hallmark pathophysiological alteration consists of involuntary, action-induced adductor spasms of the laryngeal muscles during respiration, particularly during inspiration. LABD must be distinguished from inducible laryngeal obstruction (ILO), a broader, heterogeneous condition encompassing episodic, stimulus-triggered supraglottic or glottic closure, associated with asthma, reflux, or psychological triggers, that is generally not task-specific and lacks the neurological substrate characteristic of dystonia. In contrast, LABD is a persistent, effort-dependent, neurologically driven dystonia, demonstrable by paradoxical adductor spasms on fibreoptic laryngoscopy during normal inspiration and confirmed electromyographically by paradoxical thyroarytenoid muscle activation instead of the expected inspiratory relaxation. Traditional treatments, including respiratory retraining, speech therapy, biofeedback, psychotherapy, benzodiazepines, dopamine-blocking agents, and anticholinergic drugs, have proved largely ineffective. Tracheostomy may be required in cases of severe respiratory compromise. Botulinum toxin type A (BoNT/A) injections have been reported to successfully reduce inspiratory stridor in selected patients. Here, we present three cases of LABD displaying distinct phenotypes, in which typical features were associated with involvement of extra-laryngeal cranial districts, further expanding the known phenotypic spectrum of this condition.

## 1. Introduction

Laryngeal adductor breathing dystonia (LABD) is a rare form of laryngeal dystonia characterized by inappropriate activation of laryngeal adductor muscles during respiration. Its clinical presentation may overlap with inducible laryngeal obstruction (ILO) and other functional breathing disorders, which frequently respond to respiratory retraining and speech therapy [1,2,3,4,5,6]. It is therefore essential to distinguish LABD from these functional conditions, since accurate phenotyping through multidisciplinary evaluation, laryngoscopy, and neurophysiological assessment is required to identify patients with a true dystonic disorder who may benefit from targeted botulinum toxin treatment. Indeed, while ILO and functional breathing disorders often respond well to respiratory retraining, speech therapy, and biofeedback, these approaches have been shown to be largely ineffective in patients with confirmed LABD of dystonic origin [7,8]. Therefore, accurate phenotyping through multidisciplinary evaluation, laryngoscopy, and neurophysiological assessment is essential to identify patients with a dystonic disorder who may benefit from targeted botulinum toxin treatment.

Unlike SD, patients with LABD usually have a normal voice and lack the typical speech characteristics of SD. However, as illustrated by Cases 2 and 3 in this series, LABD may coexist with other forms of laryngeal dystonia—including adductor spasmodic dysphonia—suggesting that a more precise classification as ‘laryngeal dystonia with additional breathing dystonia’ may be appropriate in such cases, reflecting the segmental rather than purely task-specific nature of the dystonic process [3,4,5,6,7,8,9].

While the main pathophysiological alteration in laryngeal dystonia (LD) consists of action-induced spasms involving the adductor or abductor laryngeal muscles, resulting in a strained or strangled voice with phonatory breaks and effortful speech in the adductor type, or intermittent breathy aphonic breaks in the abductor type, the main pathophysiological alteration in LABD is involuntary action-induced spasms of the laryngeal adductor muscles during respiration (especially inspiration) [1]. Under normal conditions, the laryngeal adductors (the thyroarytenoid (TA) and lateral cricoarytenoid (LCA) muscles) are activated during speech and inhibited during respiration [10].

In LABD patients, the lack of inhibition of these muscles during respiration leads to a distinctive clinical presentation involving inspiratory and expiratory noise during quiet respiration, which often worsens during physical activity, resulting in severe breathing difficulties [7]. A more typical clinical presentation of this condition is persistent inspiratory stridor, which usually disappears completely during sleep, only to reappear immediately upon awakening. Unlike SD, patients with LABD usually have a normal voice and lack the typical speech characteristics of SD. During fibreoptic laryngoscopy, involuntary, paradoxical adduction spasms of the vocal folds during normal inspiration can be observed, resulting in an excessively narrow glottic slit and stridor on inspiration [7]. Electromyography (EMG) of the TA muscle during inspiratory effort reveals paradoxical muscle spasm instead of the physiological TA relaxation [2]. However, in LABD, TA muscle spasms usually disappear during speech. Although LABD patients may experience varying degrees of inspiratory stridor, the resulting disability can be significant, causing daytime fatigue that interferes with normal daily activities and negatively impacts quality of life [8]. Furthermore, this condition often causes patients significant embarrassment, limiting their social life and often preventing them from working.

Traditional treatments, including respiratory retraining, speech therapy, biofeedback, psychotherapy, and the use of benzodiazepines, dopamine-blocking agents and anticholinergic drugs, have been shown to be largely ineffective in controlling symptoms in patients with confirmed dystonic LABD [7,8,11,12]. In contrast, these approaches, particularly respiratory retraining and speech therapy, are effective in patients with ILO or purely functional breathing disorders, in whom the dystonic component is absent. However, tracheostomy may be required for patients with severe respiratory impairment.

Here, we present three cases of severe LABD presenting with different phenotypes, in which the typical features of LABD were associated with involvement of the extra-laryngeal cranial district.

## 2. Case Summaries

### 2.1. Case Report 1

**Figure 1:**
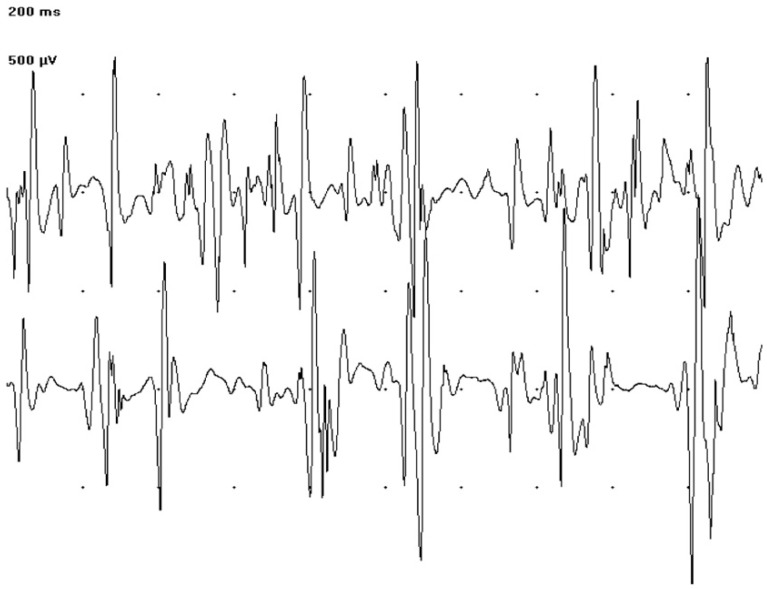
EMG recordings from right and left TA muscles. Abnormal involuntary laryngeal adductor muscle activation instead of physiological relaxation is observed on inspiration. Amp: 0.5 mV/Div; Sweep: 20 ms/Div.

A 31-year-old woman with no history of neurological disorders, head/neck trauma, pregnancy, or birth complications presented to the Neurology Department, ARNAS Garibaldi Catania (Italy) with severe breathing difficulty associated with inspiratory and expiratory noises occurring during quiet respiration and worsening during physical activity. Breathing did not significantly interfere with speech. Her symptoms had started two years prior and followed a slowly progressive course. Symptoms worsened during physical activities and disappeared during sleep; however, they showed some day-to-day variation. Before the first consultation at our clinic, the patient was examined by an ENT specialist who suspected vocal fold dysfunction. A fibreoptic endoscopy revealed normal vocal fold characteristics with normal movement during phonation. Conversely, this evaluation revealed intermittent adduction spasms of the laryngeal muscles during inspiration. These spasmodic movements were not observed during spasm-free intervals. Brain and neck CT and MRI scans were normal. Needle electromyography (EMG) of the right and left TA muscles was performed (Figure 1). Briefly, for TA muscle evaluation, a concentric EMG needle was inserted through the cricothyroid membrane, lateral to its border in a posterosuperior direction, reaching the muscle just below the infero-lateral border of the cricoid cartilage. In normal subjects, no EMG activity is observed at rest or during voluntary inspiration, whereas high-frequency activity is observed during voluntary speech, especially during vowel vocalization (e.g., “…iiiiiiii…”). The patient showed a normal EMG pattern during voluntary speech, characterized by regular activity during vocalization. Conversely, EMG revealed paradoxical TA muscle spasms during voluntary inspiration. Additionally, needle EMG of the inferior pharyngeal constrictor (IC) muscle was carried out. For IC muscle targeting, the needle was inserted 2–3 cm above the lateral aspect of the cricoid cartilage in a slightly posteromedial direction. It should be noted that at this depth and position, the needle may partially access the cricopharyngeus (CP), the lower condensation of the IC fibres, which—unlike the upper IC—normally exhibits constant tonic resting EMG activity and relaxes only briefly during swallowing. Furthermore, given its proximity to the PCA muscle, variations in CP activation may occasionally be detected during deep inspiration. These anatomical considerations are relevant to the interpretation of the EMG findings in this patient and represent a limitation of the neurophysiological assessment. Usually, the IC is activated during voluntary swallowing while remaining at rest during speech. EMG showed IC muscle phasic bursting activity during both speech and inspiration. Conversely, the IC muscle displayed physiological EMG recruitment without spasm when the patient was asked to swallow saliva or 10 mL of water.

A provisional diagnosis of LABD associated with pharyngeal dystonia was hypothesised. While the neurophysiological findings are suggestive, they are not unequivocally diagnostic of dystonia in the absence of treatment response data. Although botulinum toxin (BTX) injection into the laryngeal adductor muscles was considered, injection of the inferior constrictor pharyngis (IC) muscle was excluded due to the potential risk of dysphagia, given its important role in voluntary pharyngeal swallowing [13,14].

However, the patient refused the treatment and decided to try alternative treatments, including speech therapy, before definitively reconsidering the BTX option.

### 2.2. Case Report 2

A 54-year-old woman was admitted to the Neurology Department, ARNAS Garibaldi Catania (Italy) for severe breathing problems associated with speech difficulty. She had no history of neurological disorders, trauma, pregnancy, or birth complications. Her family history was negative for neurological and movement disorders. Upon examination, she complained of severe breathing difficulty associated with respiratory (mainly inspiratory) noises during quiet respiration that worsened during physical activity and disappeared during sleep. These symptoms were present in combination with a typical clinical presentation of adductor SD, characterized by a remarkably strained voice with phonatory breaks (especially during vocalization), noticeable changes in loudness, and effortful speech. Speech symptoms were typically relieved during whispered speech, crying, laughing, or singing. Breathing also interfered with speech. Symptoms had started more than a year prior, initially with speech difficulty and later with breathing problems, following a slow progressive course. Breathing disturbances did not improve during whispered speech, crying, laughing, or singing. Fibreoptic endoscopy revealed normal vocal fold characteristics but showed intermittent vocal fold hyperadduction during vowel phonation, which persisted as long as the vowels were sustained. Additionally, this examination revealed intermittent adduction spasms of the laryngeal muscles during voluntary inspiration. Brain and neck CT and MRI scans were normal. Needle EMG of the right and left TA muscles was performed using the previously mentioned technique. In addition, needle EMG of the right and left posterior cricoarytenoid (PCA) muscles—the main laryngeal abductors—was carried out. To target the PCA muscles, the cricoid cartilage was manually rotated medially, and the needle was percutaneously inserted lateral to the border of the cricoid cartilage in a posterior direction. Usually, no EMG activity is observed at rest; however, when the patient is asked to sniff, high-frequency EMG activity can be observed. EMG revealed TA muscle spasms during speech that disappeared at rest and during singing or whispered speech. TA spasms were also observed during inspiration. Conversely, no abnormal EMG activity was observed in the PCA muscles during speech or inspiration. LABD associated with adductor SD was hypothesized, and BTX injection into the adductor laryngeal muscles was proposed. After informed consent was obtained, incobotulinumtoxinA (dilution 200 µL) was bilaterally injected into the TA muscles (3.5 units per side) under electromyographic control. Seven days later, both the breathing difficulty (with associated respiratory noises) and the speech difficulty disappeared, with restoration of normal voice characteristics. In terms of adverse effects, the patient reported mild, transient breathy hoarseness, which began approximately three days after the injection and resolved spontaneously within ten days, without any necessary intervention. There were no reports of aspiration, dysphagia or swallowing difficulties.

Post-treatment needle EMG, performed seven days after injection, confirmed the complete disappearance of paradoxical TA muscle spasms during both voluntary inspiration and speech. No abnormal EMG activity was detected in either the right or left TA muscles at rest, during inspiration or vocalisation.

Fibreoptic endoscopy showed the resolution of the previously observed vocal fold abnormalities. The effect lasted approximately three months; specifically, it was slightly longer for SD symptoms (about 98 days) than for LABD symptoms (about 86 days). After the BTX effect wore off, the patient required further injections, which resulted in subsequent improvement.

The same doses used in the initial treatment were administered for the re-injection session: 3.5 units of incobotulinumtoxinA per side, bilaterally into the TA muscles (total dose: 7 units), under electromyographic guidance and using the same dilution (200 µL) as the initial injection. Subsequent injections were scheduled at approximately three-monthly intervals based on the observed duration of the clinical effect.

### 2.3. Case Report 3

**Figure 2:**
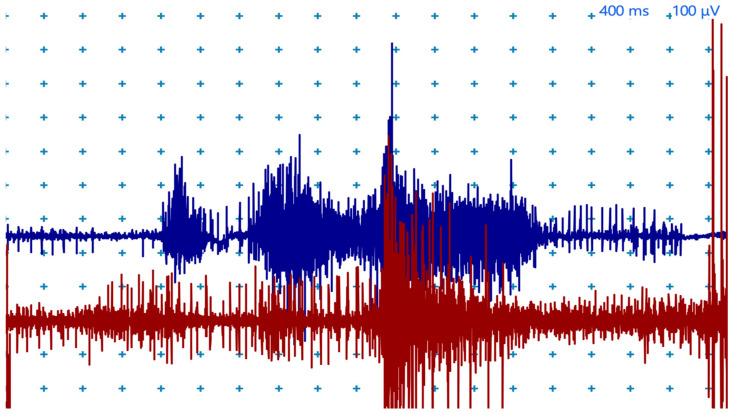
EMG recordings from TA (upper trace) and mylohyoid (lower trace) muscles show abnormal involuntary activity on inspiration in both muscles. Amp: 0.1 mV/Div; Sweep: 400 ms/Div.

A 58-year-old woman was referred to the Neurology Department at ARNAS Garibaldi Catania (Italy) for severe breathing problems, particularly during inspiration, which had a significant impact on her daily life. She also reported speech difficulty associated with anterior neck tightness and episodic dysphagia for liquids. Her family history was negative for neurological disorders. No history of neurological disease, trauma, pregnancy, or birth complications was reported. On examination, breathing difficulty associated with respiratory noises during quiet respiration was observed. Symptoms worsened during physical activity and disappeared during sleep. Speech difficulties were characterized by effortful speech with only slight changes in loudness and a mildly strained voice with phonatory breaks, occasionally associated with episodic breathy voice breaks. Speech symptoms did not improve during whispered speech, crying, laughing, or singing. She also complained of dysphagia, characterized by episodic choking (1–2 times per week) when swallowing liquids. Moreover, the patient reported a nearly constant feeling of anterior neck tightness that worsened during speech. Her symptoms had begun approximately two years prior, initially with speech difficulty and neck tightness, followed by breathing problems and dysphagia, which slowly progressed in the subsequent months. Brain and neck CT and MRI scans were normal. Fibreoptic endoscopy revealed normal vocal fold characteristics but showed abnormal contractions of the laryngeal and pharyngeal spaces during speech. Additionally, intermittent adduction spasms of the laryngeal muscles were observed during inspiration. Needle EMG of the right and left TA and PCA muscles was performed using the previously mentioned technique. In addition, needle EMG of the mylohyoid and digastric muscles was carried out. For mylohyoid muscle recordings, the needle was placed over the anterior oral floor, approximately 2 cm deep to the internal chin margin and 2 cm lateral to the midline. For digastric muscle recordings, the needle was inserted approximately 1 cm internal to the chin margin and 1 cm lateral to the midline. Usually, both muscles are active during mandibular opening [13]. TA muscle EMG recordings revealed bilateral paradoxical spasms during voluntary inspiration (Figure 2). This abnormal activity was not present during speech and was never observed in the PCA muscles. Moreover, EMG revealed continuous activity in the mylohyoid and digastric muscles during both inspiration and speech. LABD associated with hyoid dystonia was hypothesized, and BTX injection into the laryngeal adductor and submental muscles was proposed. After obtaining informed consent, incobotulinumtoxinA (dilution 200 µL) was bilaterally injected into the TA (3.5 units/side), mylohyoid (3.5 units/side), and digastric (3.5 units/side) muscles under electromyographic control. One week later, both the breathing difficulty (with associated respiratory noises) and the speech difficulty significantly improved, with restoration of normal voice characteristics. Post-treatment needle EMG, performed one week after injection, confirmed the complete disappearance of spasms in the TA, mylohyoid and digastric muscles during inspiration and speech. Specifically, no paradoxical activity was observed in the TA muscles during voluntary inspiration, and the continuous resting activity that had previously been detected in the mylohyoid and digastric muscles was no longer present. These neurophysiological findings corroborated the clinical improvement and confirmed the efficacy of the targeted BTX injections. In terms of adverse effects, the patient reported mild, transient breathy hoarseness and a slight sensation of anterior neck weakness, which began approximately four to five days after the injection. There was also a transient, mild worsening of dysphagia for liquids during the first week following the injection, consisting of occasional coughing when drinking water (two to three episodes). This was resolved completely by day ten.

Fibreoptic endoscopy showed the resolution of the previously observed vocal fold abnormalities. The effect lasted approximately three months (about 94 days). Once the BTX effect wore off, the patient required further injections, which resulted in subsequent improvement.

The same doses and injection sites used in the initial treatment were maintained for the re-injection session: 3.5 units of incobotulinumtoxinA per side, bilaterally, into the TA muscles; 3.5 units per side, bilaterally, into the mylohyoid muscles; and 3.5 units per side, bilaterally, into the digastric muscles (total dose: 21 units). This was administered under electromyographic guidance using the same dilution (200 µL) as the initial injection. This decision was based on the satisfactory clinical response and acceptable adverse effect profile observed after the first treatment. Subsequent injections were planned at approximately three-monthly intervals, consistent with the observed duration of the therapeutic effect.

## 3. Discussion

LABD is a peculiar form of respiratory disorder caused by laryngeal dystonia. This condition is considered quite rare [2,8,15]. In a study evaluating patients with different subtypes of laryngeal dystonia, only 3 out of 320 participants had LABD [2]. Klap et al. described a group of 55 SD patients treated with BTX, of whom 48 had SD, and 7 had laryngeal breathing dystonia associated with TA muscle hyperactivity [15]. These authors considered laryngeal breathing dystonia a form of laryngeal dystonia distinct from the adductor and abductor forms [2,15]. The clinical and neurophysiological findings indicated a complex laryngeal movement disorder involving inappropriate respiratory-related laryngeal muscle activation. Although there was some overlap with inducible laryngeal obstruction, the persistence of symptoms, the abnormal muscle recruitment pattern associated with them, and the response to targeted botulinum toxin treatment all supported the presence of a dystonic component with an effect duration of 6–26 weeks [7,8].

Furthermore, like SD, LABD usually presents as an isolated condition and has been considered a focal dystonia with task-specific spasms that are evident on fibreoptic endoscopy and/or EMG only during inspiratory effort. Sometimes, this condition may occur in association with other movement disorders, including cranial dystonia and atypical Parkinsonian syndromes [16,17,18]. However, the association of LABD with hyperactivity of the extra-laryngeal muscles, particularly the pharyngeal ones, has been rarely reported [7]. In a series of three LABD cases, the presence of velum, soft palate, and tongue base spasms during laryngoscopy was reported in one patient, indicating a much more widespread muscle involvement at the supraglottic/pharyngeal level [7]. In these patients, laryngo-pharyngeal spasms were observed only during respiration and did not occur during sleep [7]. Conversely, in the case series reported by Grillone et al., breathing disorders were caused prevalently by involvement at the glottic level with paradoxical vocal fold movements/hyperactivity of the TA muscles, whereas involvement of pharyngeal districts other than the laryngeal ones was not observed [7].

It is important to acknowledge that distinguishing between LABD and ILO, a functional disorder, can be challenging, particularly in cases where botulinum toxin treatment has not been administered or accepted. In Case 1, while the presence of hyperactive TA muscles on EMG is consistent with LABD, it can also be observed in ILO. However, the slowly progressive two-year course, persistence of symptoms at rest (not exclusively triggered by exercise or emotional stimuli) and synchronous involuntary recruitment of the IC muscle during inspiration—a pattern not typically reported in ILO—supported the hypothesis of a dystonic rather than purely functional disorder. However, as this patient refused BTX treatment, it was not possible to definitively demonstrate a dystonic component through treatment response. The clinical and neurophysiological findings suggested a complex form of laryngeal movement disorder characterised by inappropriate respiratory-related laryngeal muscle activation. Although overlap with ILO cannot be completely excluded, the persistence of symptoms, the abnormal muscle recruitment pattern associated with it, and the response to targeted BTX treatment—where applicable—support the presence of a dystonic component.

In the present case series, we report three patients displaying three different phenotypes of idiopathic LABD in whom the typical features of adductor breathing dystonia were associated with extra-laryngeal muscle involvement.

Detailed clinical features, instrumental findings, and BTX treatment for all cases are presented in Table 1.

In all patients, physical activity worsened breathing problems. One of them (Case 1) showed, in combination with typical LABD symptoms, the occurrence of muscle spasms in the inferior constrictor (IC) muscle during speech and inspiration, although she did not complain of dysphagia. A pharyngeal dystonia associated with LABD was hypothesized. This patient showed a clinical picture quite similar to that described in one of the patients reported by Zwirner et al. [8]. Even if a combined involvement of laryngeal and extra-laryngeal districts with muscle hyperactivity in the soft palate, velum, and tongue base muscles was observed in all patients in that study, a dorsal tilting of the epiglottis leading to complete pharyngeal closure was reported in only one of them. This closure may be explained as a mechanical condition arising from the tilting of the epiglottis rather than as a dystonia involving pharyngeal muscles. In fact, unlike our patient, in none of those three subjects was the involvement of hypopharyngeal muscles observed, and no EMG findings regarding pharyngeal muscle behaviour were reported in that case series [8]. Conversely, in our patient, EMG evaluation showed involuntary spasms in the IC muscle during inspiratory effort that were synchronous with the abnormal hyperactivation observed in the TA muscle. No EMG activity was observed in the IC muscle at rest or during phonation. The IC muscle is mainly involved in voluntary pharyngeal swallowing, being the last voluntary striated muscle to push the bolus toward the upper oesophagus [14,19]. Under normal conditions, it remains at rest during speech or respiration and is activated during voluntary swallowing. However, this patient did not complain of relevant swallowing problems, except for episodic difficulty in drinking water associated with rare coughing. Unfortunately, this patient refused BTX treatment.

Our second patient (Case 2) showed a combination of LABD with adductor SD. Although the affected areas were served by the same muscle group, this patient experienced impaired speech and respiratory function.

This association has not been reported previously. Even though speech symptoms (strained voice with phonatory breaks, changes in loudness, and effortful speech) were predominant, the clinical features of LABD were also observed. Unlike the breathing symptoms, speech symptoms were typically relieved during whispered speech, crying, laughing, or singing. BTX injection into the TA muscles reduced the severity and frequency of muscular spasms and notably improved both breathing and speech symptoms. In this case, the pathophysiological condition may be explained by a dysregulation of facilitatory/inhibitory mechanisms controlling laryngeal adductor muscles during specific tasks (speech and respiration). These muscles play a relevant role in both functions [2,4]. Although the involved districts were represented by the same muscle group, two different and independent functions (speech and respiration) were impaired in this patient. This suggests segmental cranial dystonia rather than isolated task-specific dystonia.

The association of LABD with isolated hyoid dystonia has also not been previously described.

The association of isolated hyoid dystonia with LABD has not previously been described as a standalone entity. However, Watson et al. [18] previously described breathing dystonia in the context of Meige syndrome, which was characterised by involvement of the hyoid and suprahyoid muscles alongside laryngeal breathing dystonia. In that paper, botulinum toxin treatment targeting the affected muscles was described as an effective treatment option. Case 3 differs from those reported by Watson et al. in that no features of Meige syndrome (blepharospasm or oromandibular dystonia) were present, and the breathing dystonia appeared to occur in isolation from other cranial dystonic manifestations. This suggests a distinct phenotypic presentation. Nevertheless, Watson et al.’s work [18] provides an important precedent for the association of breathing dystonia with hyoid muscle involvement, supporting the neurophysiological approach adopted in our case.

In their case series of 55 patients with hyoid dystonia, Norby et al. reported no breathing difficulties; however, on fibreoptic endoscopic examination, abnormal non-specific contractions of the laryngeal/pharyngeal space due to dystonic hyperactive hyoid muscles were observed [20]. The third patient we report (Case 3) presented with widespread impairment of different cranio-cervical districts, including the laryngeal and suprahyoid muscles, indicating a hyoid dystonia in association with LABD. Hyoid dystonia is a segmental cervical dystonia involving the platysma and suprahyoid muscles and is usually associated with other cranial dystonias, including blepharospasm and/or oromandibular dystonia. Unlike the patients described by Norby et al., breathing disturbance was a relevant clinical feature of our patient [20]. Additionally, different from typical hyoid dystonia, no association with blepharospasm or oromandibular dystonia was observed, nor was there involvement of the soft palate, velum, tongue base, or perioral muscles. A segmental cranial dystonia rather than a focal dystonia may be hypothesized in this subject.

All three patients reported in this paper shared the common feature of involuntary spasms of the laryngeal adductor muscles during inspiration, which worsened after physical activity and did not occur during speech (except for Case 2) or sleep [21]. This peculiar clinical feature differentiates this condition from more common laryngeal dystonia, where symptoms are typically observed during speech but not during respiration. Also, unlike classical laryngeal dystonia, fibreoptic endoscopy showed adduction of the true vocal folds during quiet respiration, and EMG revealed TA muscle spasms during inspiration. Instrumental evaluations disclosed no laryngeal abnormalities during speech, except for Case 2, in whom laryngeal adduction spasms were observed during both speech and respiration. All three patients showed dyspnoea that worsened with exercise. In none of them did symptoms improve during speaking, singing, yawning, laughing, or crying, as often occurs in laryngeal dystonia. However, in all patients, breathing disturbances mildly impaired speech, though only in one was this associated with true adductor spasmodic dysphonia. Both patients who were treated (cases 2 and 3) experienced mild and transient adverse effects following the injection of BTX into both sides of the larynx (bilateral TA injection), which were consistent with those reported in the literature for adductor spasmodic dysphonia. The most common adverse effect of bilateral TA injection is transient breathy hoarseness, which occurred in both cases and resolved spontaneously within ten to fourteen days [3,7]. In Case 3, a patient with pre-existing episodic dysphagia for liquids, mild transient worsening of swallowing difficulty was observed during the early post-injection period. This finding highlights the importance of careful patient selection, pre-treatment counselling and close clinical monitoring for patients with coexisting pharyngeal or suprahyoid muscle involvement, as the risk of adverse effects related to swallowing may be higher in this group than in patients with isolated laryngeal dystonia. No severe or persistent adverse effects were recorded in either patient.

## 4. Conclusions

In conclusion, this case series demonstrates that the clinical spectrum of LABD is broader and more heterogeneous than previously thought. Rather than representing three distinct, rigid phenotypes, these cases demonstrate various patterns of dystonic muscle recruitment involving the laryngeal and extralaryngeal muscles, including the pharyngeal, suprahyoid and submental muscles, which may occur together within the broader clinical spectrum of laryngeal dystonia involving the respiratory system. LABD often involves multiple anatomical regions and distinct physiological functions, even within the same muscle groups. These observations highlight the importance of not restricting LABD to a single, uniform clinical presentation and reinforce the need for a comprehensive neurophysiological evaluation to characterise the full extent of muscle involvement in each patient. Accurate clinical and neurophysiological assessment enables the targeting of botulinum toxin on an individual basis, which may provide significant symptomatic improvement. These observations further emphasise the importance of a multidisciplinary approach to evaluating patients presenting with unexplained respiratory laryngeal symptoms. These findings suggest that a comprehensive clinical and instrumental assessment of extra-laryngeal structures should be routinely performed in all patients with suspected LABD. The early identification of these diverse clinical presentations alongside classic symptoms is essential in order to prevent misdiagnosis and ineffective intervention, thus optimising the precision and efficacy of BTX treatment.

## Figures and Tables

**Table 1:** Summary of clinical and instrumental features and therapeutic effects of botulinum toxin in the reported case series of Laryngeal Adductor Breathing Dystonia (LABD).

	Disease Duration (y)	Extra Laryngeal Muscles Involvement	Fibro Endoscopy	EMG	BTX Treatment and Post-Treatment EMG
Case 1	2	Y	Vocal fold adduction and tongue base and pharyngeal activity on inspiration	Bilateral TA activity on inspiration; IPC activity on inspiration	N
Case 2	3.6	N	Vocal fold adduction in speech and inspiration	Bilateral TA spasms on both inspiration and speech	Y; disappearance of bilateral TA spasms
Case 3	3	Y	Vocal fold adduction in speech	Bilateral TA, Mylohyoid, digastric and platysma on both inspiration and speech	Y; disappearance of bilateral TA spasms

BTX = Botulinum Toxin; N = No; Y = Yes; TA = Thyroarytenoid muscle; IPC = Inferior Pharyngeal Constrictor muscle.

## Data Availability

The original contributions presented in this study are included in the article. Further inquiries can be directed to the corresponding author(s).

## Abbreviations

The following abbreviations are used in this manuscript:

BoNT/A	Botulinum toxin type A
BTX	Botulinum toxin
EMG	Electromyography
IC	Inferior pharyngeal constrictor
ILO	Inducible laryngeal obstruction
LABD	Laryngeal adductor breathing dystonia
LCA	Lateral cricoarytenoid
LD	Laryngeal dystonia
PCA	Posterior cricoarytenoid
SD	Spasmodic dysphonia
TA	Thyroarytenoid
